# Initial Riociguat Monotherapy and Transition from Sildenafil to Riociguat in Patients with Idiopathic Pulmonary Arterial Hypertension: Influence on Right Heart Remodeling and Right Ventricular–Pulmonary Arterial Coupling

**DOI:** 10.1007/s00408-018-0160-4

**Published:** 2018-09-04

**Authors:** Irina N. Taran, Anna A. Belevskaya, Marina A. Saidova, Tamila V. Martynyuk, Irina E. Chazova

**Affiliations:** 1 Department of Pulmonary Hypertension and Heart Diseases, Scientific Research Institute of Clinical Cardiology of A.L. Myasnikov, Russian Cardiology Research and Production Complex of the Ministry of Health of the Russian Federation, Moscow, Russia; 2 Ultrasonic Diagnostic Techniques Laboratory, Scientific Research Institute of Clinical Cardiology of A. L. Myasnikov, Russian Cardiology Research and Production Complex of the Ministry of Health of the Russian Federation, Moscow, Russia; 3 Department of Ultrasonic Diagnostic Techniques, Scientific Research Institute of Clinical Cardiology of A.L. Myasnikov, Russian Cardiology Research and Production Complex of the Ministry of Health of the Russian Federation, Moscow, Russia; 4 Department of Hypertension, Scientific Research Institute of Clinical Cardiology of A.L. Myasnikov, Russian Cardiology Research and Production Complex of the Ministry of Health of the Russian Federation, Moscow, Russia

**Keywords:** Idiopathic pulmonary arterial hypertension, Riociguat, Sildenafil, Echocardiography, Heart remodeling, Right ventricular–pulmonary arterial coupling

## Abstract

**Purpose:**

To evaluate the influence of riociguat on World Health Organization functional class (WHO FC), 6-min walk distance (6MWD), right heart remodeling, and right ventricular–pulmonary arterial (RV–PA) coupling in patients with idiopathic pulmonary arterial hypertension (IPAH) who are treatment-naïve or who have failed to achieve treatment goals with sildenafil therapy.

**Methods:**

Twenty patients with IPAH were enrolled: 12 had not previously received PAH-targeted therapy (treatment-naïve subgroup) and 8 had been receiving sildenafil therapy but failed to achieve treatment goals; on entering this pilot study these 8 patients were switched from sildenafil to riociguat therapy (treatment-switch subgroup). Patients received riociguat individually dose-adjusted up to a maximum of 2.5 mg three times daily. After 12 weeks, patients were assessed for WHO FC, 6MWD, right heart remodeling, and RV–PA coupling.

**Results:**

Riociguat significantly improved WHO FC in treatment-naïve patients (from 0/4/8/0 patients in WHO I/II/III/IV at baseline to 1/6/5/0 at week 12) and in treatment-switch patients (from 0/4/4/0 patients in WHO I/II/III/IV at baseline to 1/4/3/0 at week 12). Additionally, treatment-naïve and treatment-switch patients showed significant improvements at week 12 versus baseline in 6MWD (increases of + 76.8 m and + 71.6 m, respectively), RV systolic function, and RV–PA coupling.

**Conclusion:**

These results support the proven efficacy of riociguat in patients with IPAH, including treatment-naïve patients and those switching to riociguat following failure to achieve treatment goals with sildenafil, and suggest that it may be possible to delay disease progression in this patient group.

**Electronic supplementary material:**

The online version of this article (10.1007/s00408-018-0160-4) contains supplementary material, which is available to authorized users.

## Introduction

Idiopathic pulmonary arterial hypertension (IPAH; primary pulmonary arterial hypertension [PAH]) develops as a result of progressive increase in pulmonary vascular resistance (PVR). This occurs as a consequence of complex pathogenic processes in the vascular wall, including inflammation, vasoconstriction, proliferation, fibrosis, and thrombosis, resulting in the obstructive reconstruction of small pulmonary arteries and arterioles. Increasing PVR leads to right ventricular (RV) dysfunction, development of RV failure, and early death.

Before the availability of drug treatments for IPAH, median survival was 2.8 years from diagnosis, and was as low as ~ 6 months for patients with a World Health Organization functional class (WHO FC) of IV at diagnosis [1]. Current research activities are focused on studying potential therapeutic targets and developing new PAH-targeted therapies. Until recently, phosphodiesterase type 5 inhibitors (PDE5is), such as sildenafil, were the only available therapies to influence the nitric oxide (NO)–soluble guanylate cyclase (sGC)–cyclic guanosine monophosphate (cGMP) molecular pathway and decrease the rate of cGMP degradation, resulting in alleviation of IPAH. However, some patients with PAH fail to achieve treatment goals despite monotherapy or combination therapy with PDE5is [2–4].

Production of cGMP is thought to be suboptimal in patients with PAH responding inadequately to PDE5is, which has been primarily attributed to NO insufficiency [5]. This can be corrected by direct stimulation of sGC [3]. Riociguat is the first direct sGC stimulator and has a dual mode of action: sensitizing sGC to endogenous NO by stabilizing NO–sGC binding, while also directly stimulating sGC via a separate binding site, independently of NO. Via this mechanism, riociguat restores the NO–sGC–cGMP pathway, leading to increased generation of cGMP and decreased pulmonary hypertension [6–8]. Theoretically, the dual mode of action of riociguat may be a key factor for a higher potency of the drug compared with PDE5is [4].

Treatment of patients with PAH aims to achieve the following therapeutic goals: WHO FC I/II; normalization of right heart size and RV function, defined as a right atrial area (RAA) < 18 cm^2^ and the absence of pericardial effusion; mean right atrial pressure (mRAP) < 8 mmHg; cardiac index (CI) ≥ 2.5 L/min/m^2^; 6-min walking distance (6MWD) > 440 m; peak oxygen consumption (VO_2_ peak) > 15 mL/kg/min; ventilatory equivalents for carbon dioxide (VE/VCO_2_ slope) < 36; and normalization of *N*-terminal prohormone of brain natriuretic peptide (NT-proBNP) levels. The risk of 1-year mortality of patients with PAH is determined by a combination of these parameters [9, 10].

Achievement of therapeutic goals is associated with a better prognosis [10, 11]. Therefore, combination therapy is typically prescribed to patients who fail to respond adequately to initial monotherapy. However, some patients may require a more aggressive approach to achieve established therapeutic goals, e.g., upfront combination therapy at diagnosis, in accordance with current European Cardiology Society and European Respiratory Society guidelines [10, 12, 13]. A proportion of patients with PAH will fail to achieve their therapeutic goals with PDE5is, even in combination [5]. An sGC stimulator such as riociguat may be able to increase intracellular cGMP levels and achieve clinical benefits under conditions (such as NO depletion) that restrict the effectiveness of PDE5is [3, 5].

This pilot study aimed to evaluate the effect of riociguat on measures of efficacy including functional status, RV–pulmonary arterial (RV–PA) coupling, right heart remodeling, and interventricular interaction in treatment-naïve patients with IPAH and patients with IPAH failing to achieve therapeutic goals on the PDE5i sildenafil.

## Materials and Methods

This was a pilot study (registration number 8/337, cardioweb.ru) in patients with a verified diagnosis of IPAH. Patients were required to be either treatment-naïve or switching treatment from sildenafil. Patients in the treatment-switch subgroup had received sildenafil (20–80 mg three times daily [TID]) before study baseline and were assessed as having an inadequate clinical response to sildenafil therapy (see Supplementary Information for further details). The decision to transition patients to riociguat was based on the national guidelines for diagnosis and treatment of pulmonary hypertension [14].

**Fig. 1:**
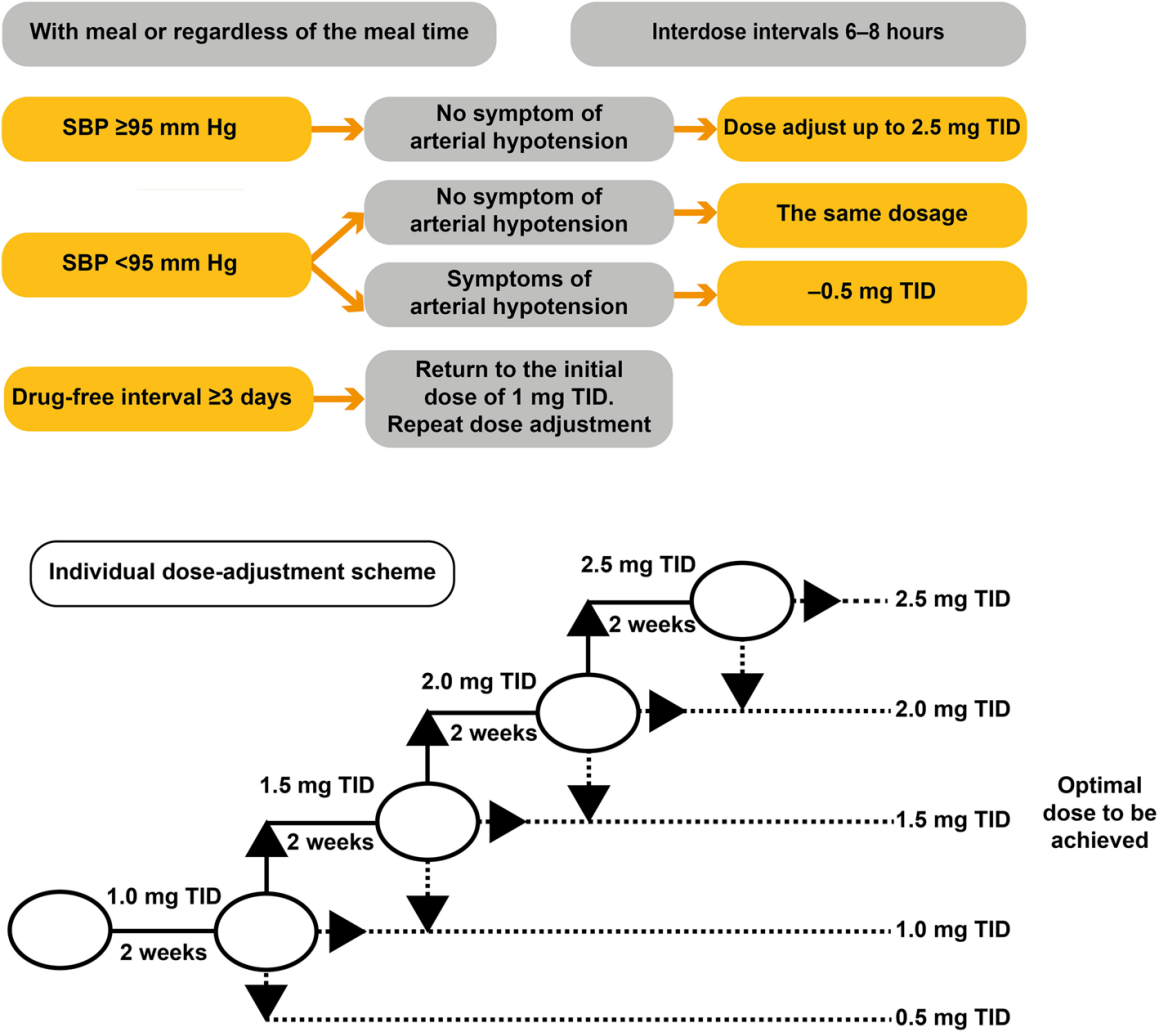
Riociguat dose-adjustment scheme. *SBP* systolic blood pressure, *TID* three times daily

Patients were hospitalized in the Department of Pulmonary Hypertension and Heart Diseases, Scientific Research Institute of Clinical Cardiology of A.L. Myasnikov, Russian Cardiology Research and Production Complex of the Ministry of Health of Russian Federation. All patients received riociguat individually dose-adjusted using the prescribed algorithm (Fig. 1), from an initial dose of 1 mg TID up to a maximum of 2.5 mg TID, in fortnightly increments of 0.5 mg, provided their systolic blood pressure was ≥ 95 mmHg and they had no symptoms of systemic hypotension. In the treatment-switch subgroup, riociguat was initiated 24 h after the final sildenafil dose.

Assessments were performed at baseline and at week 12 of riociguat treatment and included 6MWD, Borg index, oxygen desaturation index, cardiopulmonary exercise testing with assessment of VO_2_ peak, VE/VCO_2_, and echocardiography with right heart remodeling parameters and evaluation of RV–PA coupling. Right heart catheterization (RHC) was performed at baseline only to define an inadequate response to PDE5i treatment. To limit bias, assessment of 6MWD, the cardiopulmonary exercise test, and echocardiography were conducted by independent experts who were not informed about the protocol features. For details of the methodology used for these assessments, see Supplementary Information.

Statistical analysis of the data was conducted using the Statistica v. 10.0 for Windows software package (Stat Soft lnc., USA), and allowed for both parametric and non-parametric analysis. Differences between the treatment-naïve and treatment-switch subgroups were assessed using the Mann–Whitney test (*U*-test), and were considered statistically significant if *p* < 0.05. Wilcoxon non-parametric tests were used to assess post-treatment changes versus baseline. Spearman correlation coefficients were used to determine correlations between parameters. The results of the analysis are presented here as median value and interquartile range (IQR; 25th–75th percentiles).

## Results

### Baseline Characteristics and Treatment

Twenty patients with IPAH (16 females, 4 males) aged 35.7–50.0 years were enrolled between May 2016 and May 2017 (Table 1). Of these, 12 were newly started on riociguat therapy (treatment-naïve subgroup) and eight were switched to riociguat following pretreatment with sildenafil (after 21.5 ± 16.0 months; treatment-switch subgroup). At baseline, 35% and 65% of patients were in WHO FC II and WHO FC III, respectively. Overall mean 6MWD was 381.5 m (IQR 322–430). In the treatment-naïve subgroup, 67% of patients were in WHO FC III and the mean 6MWD was 381 m (IQR 305–435) at baseline. In the treatment-switch subgroup, 50% of patients were in WHO FC III before both sildenafil and riociguat therapy initiation, and mean 6MWD was 371 m (IQR 329.5–428.7) at study baseline (358 m [IQR 324–398] before starting sildenafil therapy) (Table 1).

**Table 1:** Baseline demographic and clinical characteristics, right heart remodeling, RV–PA coupling, and hemodynamic parameters

Baseline characteristic	Overall IPAH group (*n* = 20)	Treatment-naïve subgroup (*n* = 12)	Treatment-switch subgroup (*n* = 8)	*p*-value*
Age, years	43.5 [35.7–50.0]	42.5 [34.2–47.0]	47.0 [39.5–50.2]	0.3
Female, *n*	16	10	6	–
Functional status				
WHO FC I/II/III/IV, *n*	0/7/13/0	0/4/8/0	0/4/4/0	0.5
6MWD, m	381.5 [322.1–430.0]	381.5 [305.1–435.0]	371.0 [329.5–428.7]	0.9
VO_2_ peak, mL/kg/min	9.9 [7.1–12.2]	9.4 [6.7–11.4]	11.7 [7.8–13.5]	0.6
VE/VCO_2_ slope	39.0 [35.0–67.0]	48.0 [37.5–67.7]	35.1 [29.6–45.7]	0.2
Echocardiography parameters				
RA area, cm^2^	20.0 [16.0–25.7]	23.0 [16.0–27.0]	20.0 [17.0–21.5]	0.6
RVBD, cm	4.4 [4.0–4.6]	4.5 [4.1–4.7]	4.4 [3.9–4.5]	0.3
TAPSE, cm	1.7 [1.6–1.9]	1.7 [1.5–1.9]	1.7 [1.6–1.9]	0.4
DEI	1.6 [1.5–1.9]	1.7 [1.4–1.9]	1.5 [1.4–1.5]	0.7
RVFAC, %	26.5 [23.0–36.0]	23.0 [21.5–35.5]	27.0 [24.8–35.0]	0.6
SPAP, mmHg	78.0 [71.0–100.5]	78.0 [75.0–110.0]	78.0 [58.5–94.5]	0.4
mPAP, mmHg	57.0 [45.5–71.5]	61.5 [47.7–73.7]	57.0 [43.0–64.0]	0.3
RVEDV, mL	127.5 [114.7–146.7]	136.5 [130.2–156.1]	119.0 [94.8–125.2]	0.2
RVESV, mL	74.8 [66.1–91.0]	79.5 [70.0–91.0]	74.8 [67.0–88.0]	0.96
RVEF, %	32.0 [22.0–35.0]	35.0 [28.6–35.5]	32.0 [22.0–32.0]	0.4
PA Ea, mmHg/mL	0.4 [0.4–0.5]	0.4 [0.3–0.6]	0.4 [0.4–0.5]	0.2
RV E_max_, mmHg/mL	0.7 [0.6–1.0]	0.7 [0.6–1.0]	0.7 [0.5–0.8]	0.7
RV–PA coupling	0.6 [0.4–0.9]	0.6 [0.5–0.7]	0.8 [0.4–0.9]	0.5
Right heart catheterization parameters				
mPAP, mmHg	53.0 [50.0–65.0]	61.5 [51.0–68.5]	51.0 [46.5–53.0]	0.01
mRAP, mmHg	6.0 [3.7–11.8]	10.0 [5.0–14.0]	4.0 [2.0–6.0]	0.001
CI, L/min/m^2^	2.0 [1.8–2.2]	1.9 [1.6–2.0]	2.2 [2.1–2.3]	0.02
SvO_2_, %	50.5 [53.7–65.7]	59.5 [53.0–63.0]	61.0 [57.0–66.5]	0.1
PVR dyn s/cm^5^	1043.0 [761.0–1437.0]	1318.0 [956.0–1520.0]	872.0 [759.0–1056.0]	0.002

*Difference between subgroups

Data are presented as median [interquartile range] unless otherwise stated

*6MWD* 6-min walking distance, *CI* cardiac index, *DEI* diastolic eccentricity index, *IPAH* idiopathic pulmonary arterial hypertension, *mPAP* mean pulmonary artery pressure, *mRAP* mean right atrial pressure, *PA Ea* pulmonary artery effective arterial elastance, *PVR* pulmonary vascular resistance, *RA* right atrium, *RVBD* right ventricular basal diameter, *RVEDV* right ventricular end-diastolic volume, *RVEF* right ventricular ejection fraction, *RV E*_*max*_ right ventricular end-systolic elastance, *RVESV* right ventricular end-systolic volume, *RVFAC* right ventricular fractional area change, RV–PA right ventricular–pulmonary arterial coupling, *SPAP* systolic pulmonary artery pressure, *SvO*_*2*_ mixed venous oxygen saturation, *TAPSE* tricuspid annular plane systolic excursion, *VE*/*VCO*_*2*_ ventilatory equivalents for carbon dioxide, *VO*_*2*_ *peak* peak oxygen consumption, *WHO FC* World Health Organization functional class

Baseline echocardiography revealed comparable right heart dilatation, RV systolic dysfunction, impairment of interventricular interaction, and RV–PA coupling between the two subgroups (Table 1). A significant direct correlation was found between RV–PA coupling and RAA (*r* = 0.8; *p* = 0.02) and VE/VCO_2_ slope (*r* = 0.7; *p* = 0.04). Compared with treatment-naïve patients, treatment-switch patients had significantly lower baseline medians for mPAP (*p* = 0.01), mRAP (*p* = 0.001), and PVR (*p* = 0.002), as measured by RHC, and a significantly higher baseline CI (*p* = 0.02).

Analysis of clinical, functional, and hemodynamic status found that 69% of the overall population, 75% of the treatment-naïve subgroup, and 63% of the treatment-switch subgroup had a high risk of 1-year mortality; the remaining patients were all classed as intermediate risk. RAA (78%), VO_2_ peak (92%), VE/VCO_2_ slope (80%), CI (82%), and mixed venous oxygen saturation (SvO_2;_ 84%) values were the most common indicators of a high risk of 1-year mortality.

Over the 12-week study period, the median daily dose of riociguat was 6.8 mg [5.4–7.2] overall, and 6.0 mg [4.5–6.0] and 7.5 mg [6.3–7.5] in the treatment-naïve and treatment-switch subgroups, respectively.

### Overall IPAH Group Outcomes

**Fig. 2:**
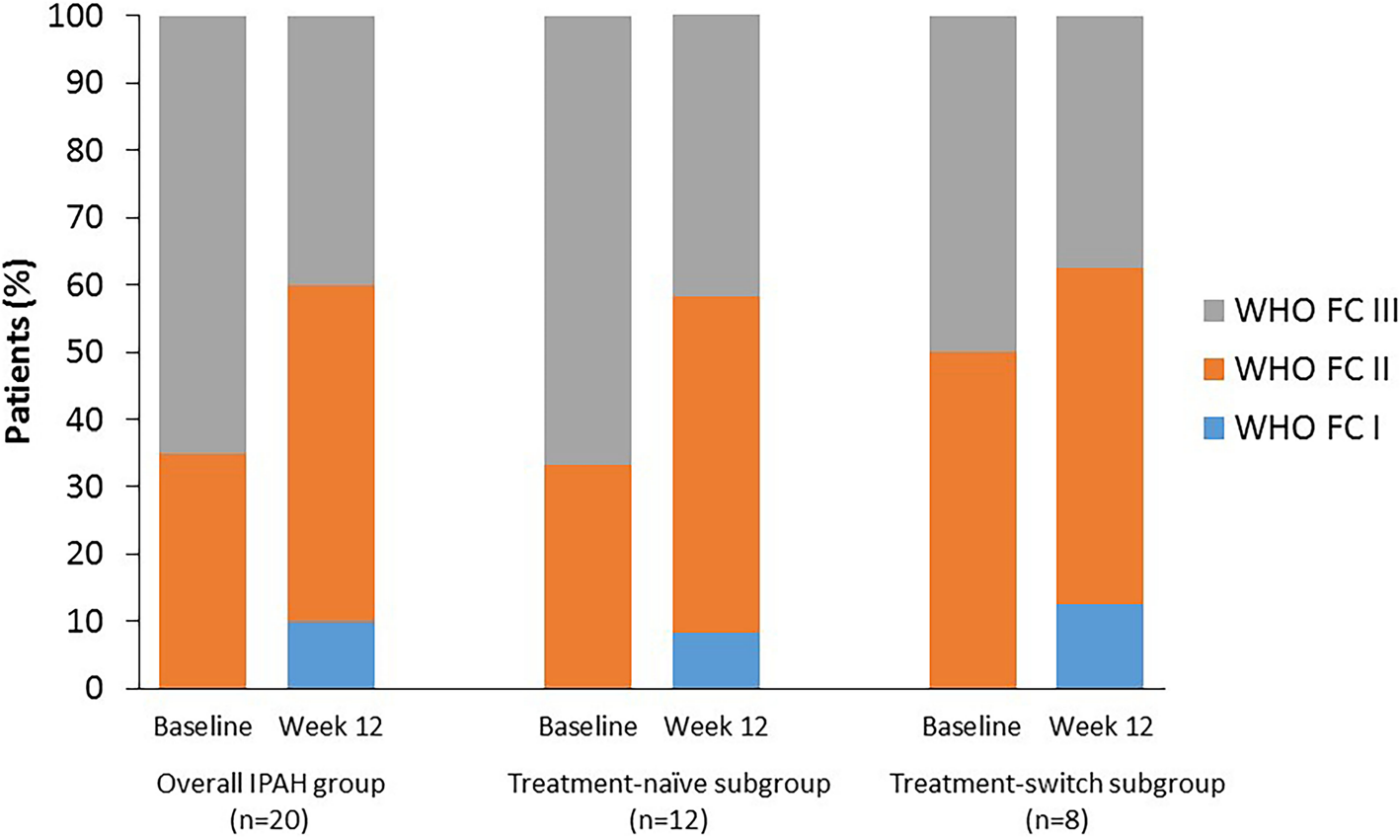
WHO FC after 12 weeks of riociguat treatment. *IPAH* idiopathic pulmonary arterial hypertension, *WHO FC* World Health Organization functional class

At week 12, patients in the overall population had achieved a significant improvement in WHO FC versus baseline (*p* = 0.001), with mean changes (∆) in 6MWD of + 76 m (*p* = 0.001), VO_2_ peak of + 2.3 mL/kg/min (*p* = 0.004), and VE/VCO_2_ slope of − 8.6 (*p* = 0.03) (Table 2; Fig. 2). Echocardiography at week 12 revealed a significant reduction in RV basal diameter (RVBD; *p* = 0.04), and decreases in mean systolic pulmonary artery pressure (SPAP) by 10.6 mmHg (*p* = 0.003) and mPAP by 6.2 mmHg (*p* = 0.007) versus baseline. Additionally, a significant increase in RV fractional area change (RVFAC; *p* = 0.04) was observed, together with reductions in RV end-diastolic volume (RVEDV; *p* = 0.003) and RV end-systolic volume (RVESV; *p* = 0.002) median values. These improvements were coupled with an improvement of the RV systolic function (as seen on three-dimensional echocardiography) (*p* = 0.04). Moreover, the RV–PA coupling value significantly decreased (*p* = 0.02) due to a decrease in PA effective arterial elastance (Ea) (*p* = 0.03) and an increase in RV end-systolic elastance (RV E_max_; *p* = 0.03) (Table 2).

**Table 2:** Functional status and echocardiographic parameters at week 12, and mean change from baseline to week 12, of riociguat treatment

	Overall IPAH group			Treatment-naïve subgroup			Treatment-switch subgroup			*p*-value (differences in mean ∆ between subgroups at week 12)
Parameter	Week 12	Change from baseline to week 12	*p*-value (week 12 versus baseline)	Week 12	Change from baseline to week 12	*p*-value (week 12 versus baseline)	Week 12	Change from baseline to week 12	*p*-value (week 12 versus baseline)	
Functional status										
WHO FC I/II/III/IV, n	2/10/8/0	–	0.001	1/6/5/0	–	0.006	1/4/3/0	–	0.02	–
6MWD, m	458.0 [370.0–480.0]	76.0	0.001	458.0 [369.0–506.0]	76.8	0.01	479.0 [424.0–486.0]	71.6	0.03	0.23
VO_2_ peak, mL/kg/min	11.1 [9.7–13.2]	2.3	0.004	10.3 [9.7–13.4]	2.7	0.02	11.9 [10.0–12.9]	1.3	0.049	0.98
VE/VCO_2_ slope	36.9 [33.0–40.0]	–8.6	0.03	37.5 [33.8–40.6]	–11.2	0.1	30.1 [28.7–32.0]	–4.8	0.04	0.03
Echocardiography parameters										
RA area, cm^2^	20.0 [18.0–23.1]	–0.46	0.5	22.0 [18.0–23.9]	–0.34	0.6	19.0 [17.0–21.0]	–0.60	0.2	0.07
RVBD, cm	4.2 [3.7–4.5]	–0.15	0.04	4.2 [4.0–4.3]	–0.20	0.03	4.2 [3.7–4.5]	–0.03	0.1	0.04
TAPSE, cm	1.8 [1.7–2.0]	0.10	0.1	1.7 [1.7–1.9]	0.13	0.4	1.8 [1.8–3.0]	0.06	0.1	0.09
DEI	1.6 [1.1–1.9]	–0.15	0.1	1.7 [1.5–1.9]	–0.14	0.3	1.1 [1.1–1.3]	–0.15	0.1	0.47
RVFAC, %	30.0 [29.0–35.0]	5.6	0.04	32.5 [29.0–37.0]	8.2	0.002	30.0 [29.0–30.0]	3.7	0.8	0.04
SPAP, mmHg	77.0 [63.5–91.0]	–10.6	0.003	77.0 [75.0–95.0]	–12.0	0.03	70.0 [58.5–84.5]	–7.5	0.02	0.08
mPAP, mmHg	48.0 [45.0–58.0]	–6.2	0.007	44.0 [36.0–52.0]	–6.3	0.007	47.0 [46.0–52.5]	–5.8	0.007	0.20
RVEDV, mL	119.0 [111.1–137.0]	–11.2	0.003	125.3 [115.9–140.1]	–16.9	0.005	114.0 [98.0–119.5]	–10.6	0.05	0.06
RVESV, mL	70.8 [61.9–80.5]	–4.4	0.002	72.6 [65.6–96.6]	–3.7	0.03	63.5 [62.2–83.5]	–4.9	0.1	0.34
RVEF, %	40.8 [31.9–42.9]	3.8	0.04	40.8 [31.9–42.9]	3.2	0.03	38.0 [34.5–41.0]	4.7	0.049	0.58
PA Ea, mmHg/mL	0.4 [0.2–0.4]	–0.06	0.03	0.4 [0.4–0.5]	–0.006	0.04	0.2 [0.2–0.2]	–0.20	0.02	0.06
RV E_max_, mmHg/mL	0.8 [0.7–1.0]	0.06	0.03	0.9 [0.7–1.0]	0.05	0.048	0.7 [0.5–0.9]	0.08	0.046	0.68
RV–PA coupling	0.5 [0.3–0.6]	–0.06	0.02	0.5 [0.5–0.6]	–0.03	0.048	0.4 [0.3–0.7]	–0.19	0.01	0.06

Data are presented as median [interquartile range] unless otherwise stated

*6MWD* 6-min walking distance, *DEI* diastolic eccentricity index, *IPAH* idiopathic pulmonary arterial hypertension, *mPAP* mean pulmonary artery pressure, *PA Ea* pulmonary artery effective arterial elastance, *RA* right atrium, *RVBD* right ventricular basal diameter, *RVEDV* right ventricular end-diastolic volume, *RVEF* right ventricular ejection fraction, *RV E*_*max*_ right ventricular end-systolic elastance, *RVESV* right ventricular end-systolic volume, *RVFAC* right ventricular fractional area change, *RV–PA* right ventricular–pulmonary arterial coupling, *SPAP* systolic pulmonary artery pressure, *TAPSE* tricuspid annular plane systolic excursion, *VE*/*VCO*_*2*_ ventilatory equivalents for carbon dioxide, *VO*_*2*_ *peak* peak oxygen consumption, *WHO FC* World Health Organization functional class

### Subgroup Outcomes

At week 12, both the treatment-naïve and treatment-switch subgroups achieved significant improvements in WHO FC (*p* = 0.006 and *p* = 0.02, respectively) and 6MWD (mean ∆: + 76.8 m [*p* = 0.01] and + 71.6 m [*p* = 0.03], respectively). Additionally, significant improvements were seen in VO_2_ peak in both the treatment-naïve and treatment-switch subgroups (*p* = 0.02 and *p* = 0.049, respectively). A decrease in VE/VCO_2_ slope at week 12 versus baseline was seen in both the treatment-naïve and treatment-switch subgroups, but only reached statistical significance in treatment-switch patients (*p* = 0.1 and *p* = 0.04, respectively) (Table 2).

Echocardiography at week 12 demonstrated significant reductions from baseline in SPAP and mPAP in both the treatment-naïve subgroup (*p* = 0.03 and *p* = 0.007, respectively) and the treatment-switch subgroup (*p* = 0.02 and *p* = 0.007, respectively). A significant reduction in RVBD (*p* = 0.03) was also observed in treatment-naïve patients. Improvement in RV systolic function was seen in the treatment-naïve subgroup (RVFAC according to two-dimensional echocardiography, *p* = 0.002; and three-dimensional echocardiography, *p* = 0.03), and also in the treatment-switch subgroup according to three-dimensional echocardiography (*p* = 0.049). The RV–PA coupling parameter decreased in both the treatment-naïve and treatment-switch subgroups (*p* = 0.048 and *p* = 0.01, respectively) due to PA Ea decrease (*p* = 0.04 and *p* = 0.02, respectively) and RV E_max_ increase (*p* = 0.048 and *p* = 0.046, respectively) (Table 2).

A between-group comparison of mean changes after 12 weeks of riociguat treatment revealed significant differences between the two subgroups in ∆VE/VCO_2_ slope (*p* = 0.03), ∆RVBD (*p* = 0.04), and ∆RVFAC (*p* = 0.04), all favoring the treatment-naïve subgroup (Table 2).

Two patients (both treatment-naïve at baseline) remained at high risk of 1-year mortality after 12 weeks of riociguat therapy [10]. Dose adjustment to > 1.5 mg TID was not possible in these 2 patients because of systemic hypotension, and a second PAH-specific drug was prescribed in addition to riociguat.

### Safety

The most common adverse events were systemic hypotension (10%, *n* = 2), tachycardia (10%, *n* = 2), and exacerbation of gastroesophageal reflux disease symptoms (10%, *n* = 2). No adverse events required discontinuation of riociguat treatment.

## Discussion

Convincing evidence of the efficacy of and tolerability of riociguat in patients with PAH has been obtained previously from the well-designed randomized, placebo-controlled phase III trial, PATENT-1 (Pulmonary Arterial Hypertension Soluble Guanylate Сyclase-Stimulator Trial 1). In PATENT-1, riociguat treatment significantly increased exercise capacity and improved a range of secondary endpoints, including hemodynamic variables, WHO FC, and time to clinical deterioration [15, 16]. The PATENT-2 long-term open-label extension trial demonstrated sustained benefits with riociguat treatment at 2 years, with an average increase of 47 m in 6MWD versus baseline, a 2-year survival rate of 93%, and clinical worsening-free survival rate of 79% [17, 18].

Transition from a PDE5i to riociguat was tested in a prospective, international, multicenter, open, non-comparative, phase IIIb trial, RESPITE (Riociguat Clinical Effects Studied in Patients with Insufficient Treatment Response to PDE5is). RESPITE evaluated the efficacy and safety of transition from sildenafil or tadalafil to riociguat in 61 patients with PAH who failed to meet treatment goals on PDE5is. The trial was designed with an 8-week riociguat dose-adjustment period followed by a 16-week period of stable treatment at a maintenance dose of riociguat up to 2.5 mg TID [3, 4]. At week 24, 52% of patients had improved from WHO FC III to WHO FC II (2% improved to WHO FC I). Other improvements included a significant increase from baseline to week 24 in mean ± SD 6MWD by + 31 ± 63 m, a decrease in PVR by − 103 ± 296 dyn s/cm^5^, an increase in CI by + 0.3 ± 0.5 L/min/m^2^, and a decrease in NT-proBNP level by − 347 ± 1235 pg/mL [4]. Currently the published evidence around transitioning patients from PDE5i to riociguat is limited to RESPITE and some case study data, while a randomized controlled trial is currently under way (Riociguat rEplacing PDE-5i Therapy evaLuated Against Continued PDE-5i thErapy [REPLACE; NCT02891850]). The findings from our pilot study show improvements in functional status and good tolerability in a small group of patients who transitioned from PDE5i to riociguat. These data add to the current pool of evidence for the use of transition as a treatment option and help to build the clinical knowledge around this area.

Cardiovascular coupling has been of special research interest in recent years. Regulation of systemic blood pressure, increase in cardiac output, and ability to respond adequately to increased heart rate all depend not only on cardiac function but also on the condition of the cardiovascular system, more precisely, on left ventricular-aortic coupling. Maintaining normal cardiovascular coupling improves the mechanic and metabolic efficiency of the cardiovascular system. Therefore, cardiovascular coupling parameters may be regarded as key factors in maintaining adequate cardiovascular performance [19, 20].

Currently, only limited information is available about the effect of PAH-specific therapy on right heart remodeling and RV–PA coupling. As RV function is a key determinant of both functional status and survival of patients with severe PAH, this is an important potential outcome for evaluating the beneficial effects of PAH-specific therapy.

Pulmonary hypertension progression leads to pressure overload and RV failure, which are followed by marked impairment in cardiovascular coupling [21]. A pilot study of interventricular interaction and RV–PA coupling has been performed in patients with IPAH of varying disease severities in the Scientific Research Institute of Clinical Cardiology of A.L. Myasnikov, Russian Cardiology Research and Production Complex. The study found that PA Ea and RV–PA coupling were significantly higher in patients with IPAH categorized as WHO FC III/IV versus those who were WHO FC I/II. A correlation analysis revealed a significant direct relationship between RV–PA coupling and RAA and a significant inverse relationship between both PA Ea and RV–PA coupling and systolic function parameters (RV ejection fraction, fractional area change, and tricuspid annular plane systolic excursion) [22].

No prior study has reported on the effects of riociguat on right heart remodeling and RV–PA coupling in either treatment-naïve patients with IPAH or patients with IPAH who have failed to achieve treatment goals with sildenafil. Our pilot study indicates that riociguat treatment may result in significant improvements in RV systolic function and decreases in RV–PA coupling for patients with IPAH, across both treatment-naïve and treatment-switch subgroups. These observations suggest that riociguat is highly effective for the treatment of patients with IPAH, and delaying disease progression in these patients may be possible.

This pilot study evaluated right heart remodeling parameters, as well as the RV–PA coupling, and their clinical and predictive value in routine practice requires further research. However, these parameters provide important information about the dynamic evaluation of the effectiveness of therapy in patients with IPAH.

This study has limitations: this was not a randomized, controlled study; the sample size was relatively small; and some clinical and laboratory data for some patient visits were not available at the time of data analysis (e.g., BNP, NT-proBNP). Additionally, RHC was not performed after 12 weeks as non-invasive criteria were considered adequate to assess response to treatment after this short period. Although the study was non-blinded, bias was limited by using independent experts who were unaware of the treatment allocations to conduct patient assessments.

In conclusion, riociguat treatment demonstrated a positive effect on functional status, RV–PA coupling, and right heart remodeling in treatment-naïve patients with IPAH and in patients with IPAH who failed to achieve treatment goals with sildenafil and who were switched to riociguat, and was well tolerated. This suggests that transitioning from sildenafil to riociguat may be an effective therapeutic strategy in patients with IPAH who fail to have an adequate treatment response to PDE5i. However, further large-scale, randomized, controlled studies are needed to confirm these initial findings.

## Electronic supplementary material

Below is the link to the electronic supplementary material.

Supplementary material 1 (DOCX 15 KB)
