# Supplementary material for: Initial Riociguat Monotherapy and Transition from Sildenafil to Riociguat in Patients with Idiopathic Pulmonary Arterial Hypertension: Influence on Right Heart Remodeling and Right Ventricular–Pulmonary Arterial Coupling
Source: Lung. 2018 Sep 4;196(6):745–53. doi: 10.1007/s00408-018-0160-4 (PMC6244659; doi:10.1007/s00408-018-0160-4)
Supplement: Supplementary file 1 — Supplementary material 1 (DOCX 15 KB) [file 408_2018_160_MOESM1_ESM.docx]

# Supplementary Information

**Materials and Methods**

Patients with inadequate response to sildenafil therapy had at least one of the following criteria at baseline: World Health Organization functional class (WHO FC) III/IV; 6-minute walking distance (6MWD) <440 m; peak oxygen consumption (VO_2_ peak) <15 mL/kg/min; ventilatory equivalents for carbon dioxide (VE/VCO_2_ slope) >44.9; and inadequate hemodynamic parameters assessed by right heart catheterization (RHC) (mean pulmonary artery pressure [mPAP] >30 mmHg; mean right atrial pressure [mRAP] >8 mmHg; and cardiac index [CI] <2.5 L/min/m^2^). The presence of at least one of the above criteria indicated the need for optimizing therapy and the decision to transition patients to riociguat was made by a multidisciplinary group that included both an independent clinician and a member of the study team. For patients in the treatment-switch subgroup, more weight was given to cardiopulmonary exercise testing parameters (especially VO_2_ peak) and RHC parameters (especially CI) than the other clinical response criteria when making the decision to switch treatment.

Cardiopulmonary exercise testing was performed on the CARDIOVIT CS-200 Ergo-Spiro multifunctional exercise testing system (SCHILLER, Switzerland) with a Power Cube gas analyzer (Ganshorn, Germany) using a breath-by-breath gas analysis method to evaluate gas exchange for >10 intervals. In accordance with the protocol, the testing procedure included a 3-min rest period followed by pedaling at 60 rpm for 5–7 min at a gradual load rise from 5 to 25 W (up to the maximum tolerated load), followed by a 5-min rest. Measurements of oxygen consumption (VO_2_) and exhaled carbon dioxide (VCO_2_) were performed using the breath-by-breath method. VO_2_ peak was defined as the highest VO_2_ value measured in the last minute of exposure to load.

Transthoracic echocardiography was performed on the Vivid E9 expert-class ultrasonic scanner (GE Healthcare, USA) using the M5S-D sensor for two-dimensional imaging and 4V-D matrix sensor for three-dimensional imaging. Phases of the cardiac cycle were synchronized with electrocardiography (ECG). Systolic pressure in the pulmonary artery (SPAP) was calculated by the formula: SPAP=maxSTRG + RAP, where maxSTRG is maximal systolic tricuspid regurgitation pressure gradient [1,2]. The value of mPAP was calculated as follows: mPAP=mSTRG + RAP, where mSTRG is mean systolic tricuspid regurgitation pressure gradient [3]. RAP was determined based on the inferior vena cava diameter and its collapse on inspiration. Pulmonary artery wedge pressure (PAWP) was calculated using the Nagueh formula: PAWP=1.24 ×Е/E’ + 1.9, where E is the mitral peak velocity of early filling, as measured by pulsed wave Doppler, and E’ is the early diastolic mitral annular velocity, as measured by tissue myocardial Doppler [4].

The apical 4-chamber views on echocardiography were used to evaluate the right ventricular (RV) systolic function in the three-dimensional mode. The image was then transferred to the EchoPac PC work station (GE Healthcare, USA), which includes built-in special TomTec software for calculating RV end-systolic volume (RVESV), RV end-diastolic volume (RVEDV), and RV ejection fraction (RVEF). Tricuspid annular plane systolic excursion (TAPSE) and fractional area change (FAC) values were used to evaluate the RV systolic function in the two-dimensional mode. Stroke volume (SV) was calculated by the formula: SV = πr^2^×VTI, where r is the radius (1/2 of diameter) of the RV outflow tract (RVOT) and VTI is the RVOT velocity time integral. Diastolic eccentricity index (DEI) was used as a measure of interventricular interaction, and was defined as the ratio of mutually perpendicular left ventricular (LV) diameters measured from the parasternal view along the short LV axis at the level of papillary muscles [5].

The RV–pulmonary arterial (RV-PA) coupling was calculated as Ea:E_max_ ratio, where Ea is the effective arterial elastance and E_max_ is the ventricular end-systolic elastance [6,7]. Effective arterial elastance was calculated as follows: Ea = (mPAP – PAWP)/SV and ventricular end-systolic elastance was calculated by the formula: E_max_ = mPAP/ESV, where ESV is the RVESV [6].

Patients enrolled in the pilot study underwent RHC at baseline (control RHC is recommended at Week 24 of riociguat therapy). The Allura Xper FD10 cardiovascular X-ray system (Philips, the Netherlands) was used to perform RHC. The procedure included both direct manometry of RAP, PAP, and PAWP using a Swan–Ganz catheter, and the calculation of mPAP and pulmonary vascular resistance.

**Supplementary References**

1. Hatle L, Angelsen BA, Tromsdal A (1981) Non-invasive estimation of pulmonary artery systolic pressure with Doppler ultrasound. Br Heart J 45(2):157–165

2. Yock PG, Popp RL (1984) Noninvasive estimation of right ventricular systolic pressure by Doppler ultrasound in patients with tricuspid regurgitation. Circulation 70(4):657–662

3. Aduen JF, Castello R, Lozano MM, Hepler GN, Keller CA, Alvarez F, Safford RE, Crook JE, Heckman MG, Burger CD (2009) An alternative echocardiographic method to estimate mean pulmonary artery pressure: diagnostic and clinical implications. J Am Soc Echocardiogr 22(7):814–819. doi:10.1016/j.echo.2009.04.007

4. Nagueh SF, Middleton KJ, Kopelen HA, Zoghbi WA, Quinones MA (1997) Doppler tissue imaging: a noninvasive technique for evaluation of left ventricular relaxation and estimation of filling pressures. J Am Coll Cardiol 30(6):1527–1533

5. Ryan T, Petrovic O, Dillon JC, Feigenbaum H, Conley MJ, Armstrong WF (1985) An echocardiographic index for separation of right ventricular volume and pressure overload. J Am Coll Cardiol 5(4):918–927

6. Sanz J, Garcia-Alvarez A, Fernandez-Friera L, Nair A, Mirelis JG, Sawit ST, Pinney S, Fuster V (2012) Right ventriculo-arterial coupling in pulmonary hypertension: a magnetic resonance study. Heart 98(3):238–243. doi:10.1136/heartjnl-2011-300462

7. Kass DA (2002) Age-related changes in venticular-arterial coupling: pathophysiologic implications. Heart Fail Rev 7(1):51–62
